# LC-MS Based Sphingolipidomic Study on A2780 Human Ovarian Cancer Cell Line and its Taxol-resistant Strain

**DOI:** 10.1038/srep34684

**Published:** 2016-10-05

**Authors:** Hao Huang, Tian-Tian Tong, Lee-Fong Yau, Cheng-Yu Chen, Jia-Ning Mi, Jing-Rong Wang, Zhi-Hong Jiang

**Affiliations:** 1 State Key Laboratory of Quality Research in Chinese Medicine, Macau Institute for Applied Research in Medicine and Health, Macau University of Science and Technology, Taipa, Macau, China; 2 College of Pharmacy, Gannan Medical University, Ganzhou 341000, China

## Abstract

Drug resistance elicited by cancer cells continue to cause huge problems world-wide, for example, tens of thousands of patients are suffering from taxol-resistant human ovarian cancer. However, its biochemical mechanisms remain unclear. Sphingolipid metabolic dysregulation has been increasingly regarded as one of the drug-resistant mechanisms for various cancers, which in turn provides potential targets for overcoming the resistance. In the current study, a well-established LC-MS based sphingolipidomic approach was applied to investigate the sphingolipid metabolism of A2780 and taxol-resistant A2780 (A2780T) human ovarian cancer cell lines. 102 sphingolipids (SPLs) were identified based on accurate mass and characteristic fragment ions, among which 12 species have not been reported previously. 89 were further quantitatively analyzed by using multiple reaction monitoring technique. Multivariate analysis revealed that the levels of 52 sphingolipids significantly altered in A2780T cells comparing to those of A2780 cells. These alterations revealed an overall increase of sphingomyelin levels and significant decrease of ceramides, hexosylceramides and lactosylceramides, which concomitantly indicated a deviated SPL metabolism in A2780T. This is the most comprehensive sphingolipidomic analysis of A2780 and A2780T, which investigated significantly changed sphingolipid profile in taxol-resistant cancer cells. The aberrant sphingolipid metabolism in A2780T could be one of the mechanisms of taxol-resistance.

Ovarian cancer is the most aggressive gynecologic cancer and thus a leading cause of cancer-related mortality in women worldwide^1^. At present, the most effective strategy for ovarian cancer is combination therapy based on cytoreductive surgery and chemotherapy with taxanes (e.g. taxol), but intrinsic or acquired tumor chemoresistance remains the most important clinical problem and a major obstacle to a successful therapy^2^. According to a systematic literature review, 69 of the total 137 acquired drug-resistant cell lines were resistant to taxol^3^. Seventy-five percent of ovarian cancer patients initially respond to platinum or taxane based chemotherapy; however, most of them eventually develop chemotherapy resistance^4^. Many factors can lead to drug resistance, including increased drug efflux, drug inactivation, alterations in drug target, processing of drug-induced damage, and evasion of apoptosis^5^. Mechanisms including overexpression of drug resistant associated proteins^6^ and activation of some signaling pathways^7^ have been implicated in resistance to taxol, but the overall molecular mechanisms of taxol resistance still need further elucidation.

Sphingolipids (SPLs) are a kind of membrane and intracellular lipids that typically play structural roles and act as signaling molecules and/or modulators of signaling pathways associated with cell survival^8^. Besides the most widely studied bioactive SPL - ceramide, the relationship between cancer and other SPL has been extensively studied, including sphingosine 1-phosphate (S1P)^9^, glucosylceramide (GluCer)^10^, sphingosine and C1P^11^. Growing evidence showed that sphingolipids are deeply involved in the regulation of apoptosis as well as the apoptosis resistance that is displayed by cancer cells^12^. Qualitative and quantitative assessment of SPLs could reveal novel biomarkers for early diagnosis of cancer^13^.

There are several studies focused on the sphingolipidomics of A2780 Human Ovarian Cancer cell line^14^,^15^, as well as its fenretinide-resistant^16^ and multidrug-resistant strains^17^,^18^. Valsecchi M *et al*. have characterized the sphingolipidomes in *N*-(4-hydroxyphenyl)retinamide (4-HPR) and 4-oxo-*N*-(4-hydroxyphenyl)retinamide (4-oxo-4-HPR) treated A2780 cells by ESI-MS, revealed that the two drugs differentially affect the early steps of SPL synthesis^19^. In 4-HPR resistant A2780 cells (A2780/HPR), a remarkable alteration of sphingolipid metabolism with respect to both of the parental sensitive A2780 cells and 2780AD cells has been revealed^20^. Increasing evidence suggests the change of SPL metabolism can be (one of) the crucial mechanism of drug resistance in A2780 cells. However, till now, there is no sphingolipidomic study on taxol resistant A2780 cells (named as A2780T, TA2780, A2780/Taxol, or A2780/PTX in literature). Therefore, a comprehensive sphingolipidomic study is required for elucidating the mechanisms underlying the resistance of A2780T cells to taxol treatment.

In the current study, SPLs in A2780 and A2780T were comprehensively profiled and quantitatively determined by using a well-established LC-MS approach developed in our lab^21^. It appears to be a promising tool for viewing overall sphingolipidomic difference between taxol-sensitive and -resistant strain of A2780.

## Results

### Comprehensive identification of sphingolipids in A2780 cells

Duplicate analyses of pooled samples of A2780 and A2780T cells (QC samples) were carried out to achieve comprehensive profiling of SPLs in these two cell lines. Ultra-high performance liquid chromatography coupled with Q-TOF mass spectrometry (UHPLC-Q-TOF MS) is an effective and sensitive analytical tool to separate and identify SPLs in a complex mixture. By integrating the high efficient separation offered by UHPLC, high-resolution mass spectrum obtained by MS and MS/MS on Q-TOF, as well as comparing the data with those of reference standards and searching against our personal database, totally 102 SPLs have been identified in the pooled samples, among which six ceramides (d18:1/17:3; d18:1/15:3(OH); d18:1/14:3(OH); d18:2/23:1; d18:0/18:3 and d17:0/13:0(OH)), two ceramide-1-phosphates (d18:1/19:0(OH) and d18:1/12:2), one hexosylceramide (d18:1/20:1), and three sphingosines (d16:3; d15:3 and t19:2) are new SPLs. Sixty-seven out of the 102 SPLs were reported for the first time in A2780 cells.

**Figure 1:**
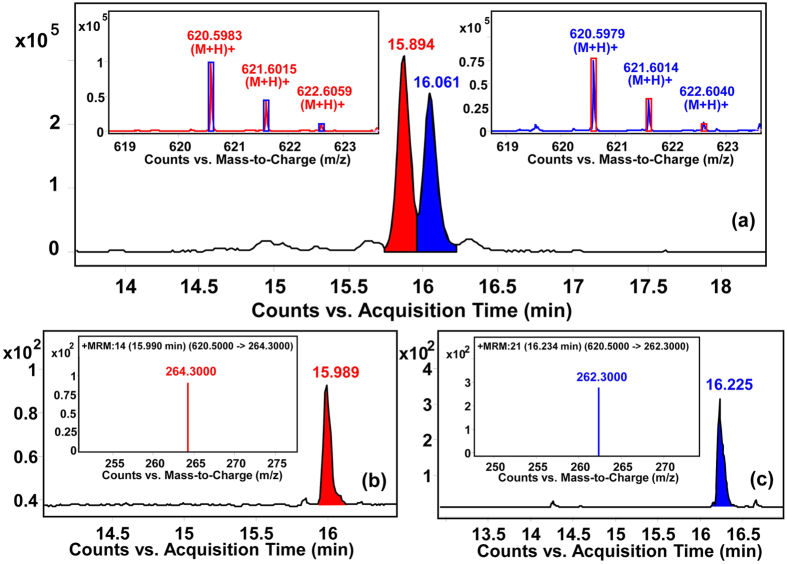
Differentiation of isomeric SPLs by targeted MS/MS. (**a**) Two peaks were observed at *m*/*z* 620.59 in extracted ion chromatogram of TOF MS. Accurate MS/MS data confirmed these peaks corresponding to Cer (d18:1/22:1) and Cer (18:2/22:0) due to the characteristic fragment at 264.3 and 262.3 respectively. In MRM mode, target ion pairs consist of the same parent ion (620.6) but different daughter ions [(**b**) 264.3 for Cer (d18:1/22:1) and (**c**) 262.3 for Cer (18:2/22:0)] were employed for the accurate quantitation.

MS signals might be masked by isomeric, isotopic or isobaric ions. For sphingolipidomic profiling of A2780, our improved sphingolipidomic approach showed great potential in differentiating isomeric and isotopic species as that have been observed in PC12 cells^21^. A major interference in the identification of SPLs is the isomeric species that have exact identical molecular elemental compositions, thus MS/MS data together with optimized separation are essential for discrimination. For instance, the extracted ion chromatogram of *m*/*z* 620.5903 at 5 ppm mass accuracy yielded two peaks at 15.894 and 16.061 min. Targeted MS/MS of *m*/*z* 620.6 at respective time points gave distinct product ions corresponding to backbone of Cer (d18:1/22:1) (*m*/*z* 264.3) and Cer (d18:2/22:0) (*m*/*z* 262.3), providing evidence for the identification of these two species (Fig. 1). The targeted ion pairs together with complete chromatographic separation also enabled subsequent quantification of such isomers by using multiple reaction monitoring (MRM) technique. Notably, 4 pairs of isomeric species (**A**_**1**_–**A**_**4**_) were clearly distinguished in our study (Table 1).

The comprehensive profiling of SPLs provided an overall “picture” of the sphingolipidome of A2780 cells. Generally, sphingomyelin (SM) is the most abundant subclass of SPLs in this cell line. Totally 43 SMs, including 31 dehydrosphingomyelins and 12 dihydrosphingomyelins (DHSMs), were identified based on exact mass and characteristic product ions obtained in targeted MS/MS experiments, 31 of which are reported for the first time in A2780 cell line. All the SMs were found to possess a C18 sphingoid base chain, with d18:1 account for the majority, comparing to the d18:0 and d18:2 backbones. The length of N-acyl chain varies from 14 to 26, and the unsaturation degree ranges from 0 to 5. Notably, the N-acyl chains of all the 12 DHSMs are fully saturated. Two highly unsaturated (total unsaturation degree no less than 4) SMs, SM (d18:1/24:3) and SM (d18:2/24:3), have been detected in A2780 cells for the first time.

In A2780 cells, 26 Cers, including 19 dehydroceramides and 7 dihydroceramides (DHCers), were identified based on the MS information and, in some cases, by comparing the retention time with that of SPLs in PC12 cells in our previous study^21^. Most Cers detected in the sample were with a d18:1 sphingoid backbone, with carbon number of N-acyl chain ranged from 14 to 24. Three dehydroceramides and 4 DHCers with a hydroxyl group on N-acyl chain have been characterized, among which 2 dehydroceramides and 1 DHCer with short N-acyl chain (carbon number less than 16) were reported for the first time to the best of our knowledge. The other 3 new Cers were species with high degree of unsaturation, for instance, Cer (d18:1/17:3), Cer (d18:2/23:1) and a new DHCer (d18:0/18:3). A notable ceramide was DHCer (d17:0/13:0(OH)), which was a very uncommon DHCer with odd carbon number sphingoid backbone.

Due to the limitation of chromatographic separation, galactosylceramide and glucosylceramide cannot be distinguished, thus these two hexose-linked ceramides were represented as HexCer. All C1P, HexCer, and lactosylceramide (LacCer) species exclusively bared a d18:1 sphingoid base backbone. The dominant HexCers and LacCers are d18:1/24:1, d18:1/24:0 and d18:1/16:0. Two novel C1Ps, *i.e.* C1P (d18:1/19:0(OH)) and C1P (d18:1/12:2), were identified in A2780. The former one has an N-acyl chain with odd carbon number and a hydroxyl group, while the latter one has two degrees of unsaturation on the N-acyl fatty chain.

Eighteen sphingoid bases with carbon number ranging from 14 to 20 were successfully identified. Short chain sphingosines with high unsaturation degree (d16:3 & d15:3) and a sphingosine with 3 hydroxyl groups (t19:2) have been discovered as uncommon species.

### Quantitative profiling of sphingolipidome in A2780 cells

**Figure 2:**
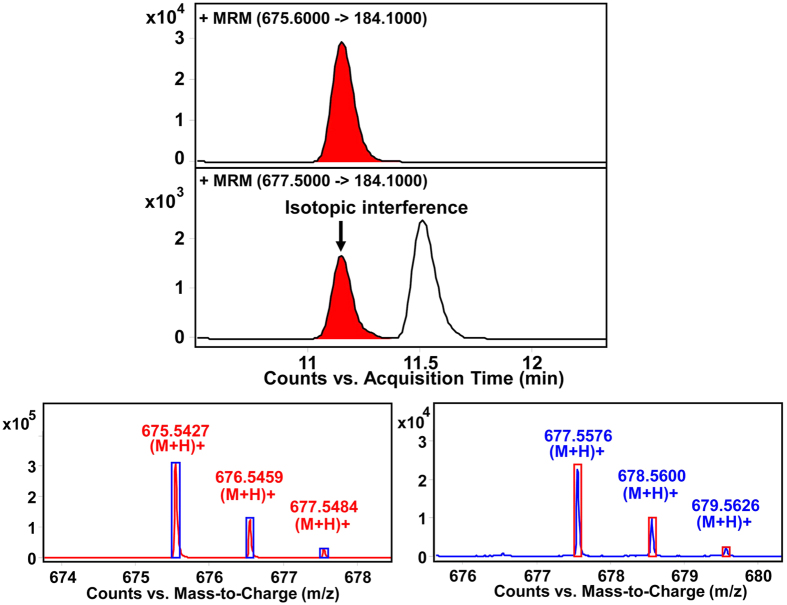
Differentiation of isotopic SPLs by accurate MS together with complete separation. (**a**) SM (d18:1/14:0) (t_R_ = 11.190 min) yields precursor ions at *m*/*z* 675.5427, 676.5459 and 677.5484. The last one is the [M + 2] isotopic ion which will interfere with the precursor ion of (**b**) SM (d18:0/14:0) (t_R_ = 11.523 min) at *m*/*z* 677.5576. If the chromatograph cannot separate the two SPLs completely, the quantification result of SM (d18:0/14:0) will be artificially high.

Comparing to routine LC-MS based approaches, UHPLC coupled with QQQ mass spectrometer in MRM mode provides more sensitive and accurate quantification with wider dynamic range of SPLs. However, the quantification of SPLs cannot be accomplished accurately in LC-MS/MS analysis with a QQQ analyzer solely, as triple-quadruple cannot distinguish isotopic/isobaric ions within 0.1 Da when selecting the precursor ions. For instance, each unsaturated SPL could generate an isotopic interference on SPLs with less degree of unsaturation as exemplified by SM (d18:1/14:0) and SM (d18:0/14:0) (Fig. 2). In our approach, based on foregoing comprehensive profiling by Q-TOF and the optimized chromatographic separation, all the structurally similar SPLs were accurately quantified with elimination of such isotopic/isobaric interference. With the optimized MRM conditions, 89 species from 9 subclasses out of 102 identified SPLs were quantified by using the UHPLC-QQQ MS method. It was found that A2780 and A2780T share most common sphingolipid molecules, except for HexCer (d18:1/20:1) which is only present in A2780. The amounts of SPLs were measured by using the internal standards previously mentioned, duplicate measurements for each sample yielded consistent results in all cases.

**Figure 3:**
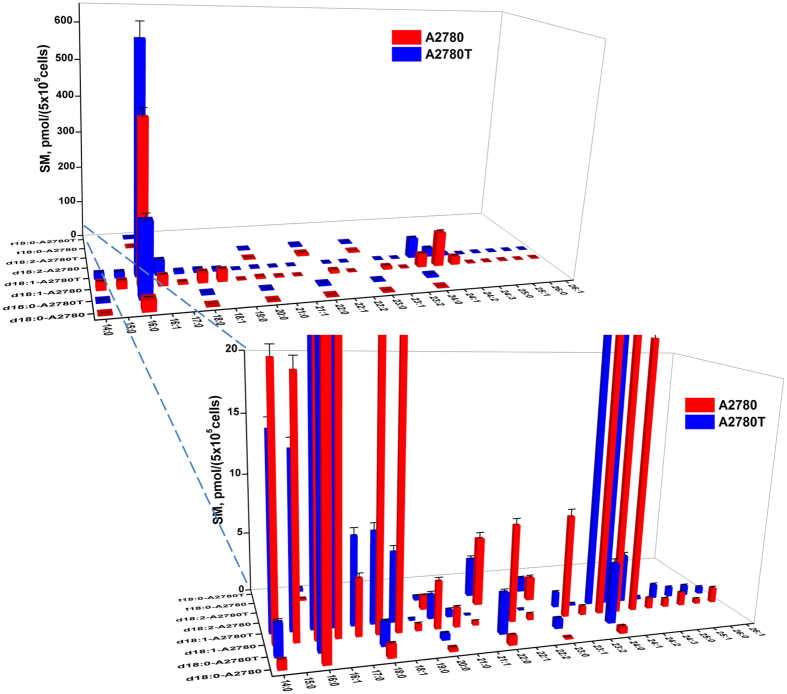
Content of marker sphingomyelins in A2780 and A2780T. The horizontal and depth axes represent the composition of fatty acid acyl chain and sphingoid base backbone chain, respectively.

The quantitative results showed that SMs take the highest proportion of all the SPLs, among which SMs with C18 sphingoid base backbone are the dominant species (Fig. 3). In A2780 cells the most dominant species are SM (d18:1/16:0) which corresponding to [M + H]^+^ at *m*/*z* 703, followed by DHSM (d18:0/16:0) (*m*/*z* 705), SM (d18:1/16:1) (*m*/*z* 701) with less relative abundance. The d18:1 SMs with C16/C18/C22/C24 N-acyl chain showed relative high levels in both A2780 and A2780T. Forty-two out of the 43 SMs were quantified except for DHSM (d18:0/25:0), whose content is lower than the limit of quantitation (LOQ).

**Figure 4:**
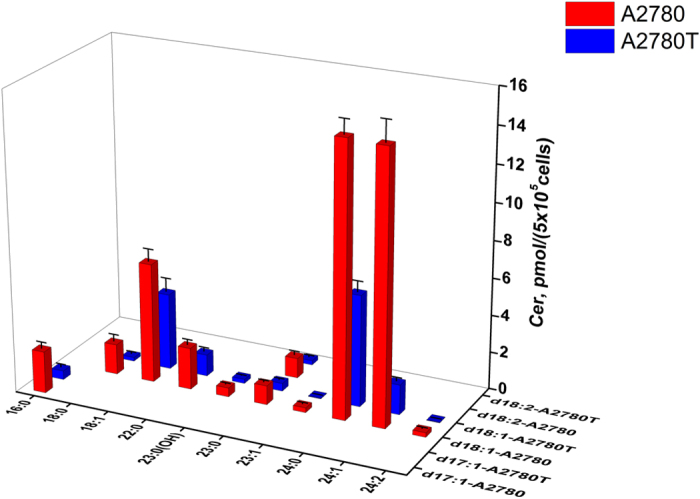
Content of marker ceramides in A2780 and A2780T. The horizontal and depth axes represent the composition of fatty acid acyl chain and sphingoid base backbone chain, respectively.

A total of 20 Cers have been quantified, but most of them are d18:1 species due to the weak intensity of d18:0 backbone fragment ions (*m*/*z* 266.4). According to the finding of Koyanagi *et al*. in tumors only the content of C16 N-acyl chain ceramide (C16-Cer) are significantly high^22^, that can explain why other DHCers cannot be quantified exactly. As shown in Fig. 4, the content of individual Cers differ dramatically (at most 500 times), for some common species like d18:1/18:1, d18:1/24:0 and d18:1/24:1, the contents are significantly higher than that of highly unsaturated species d18:1/24:2. In general, the amounts of Cers are significantly higher in A2780 than those in A2780T.

**Figure 5:**
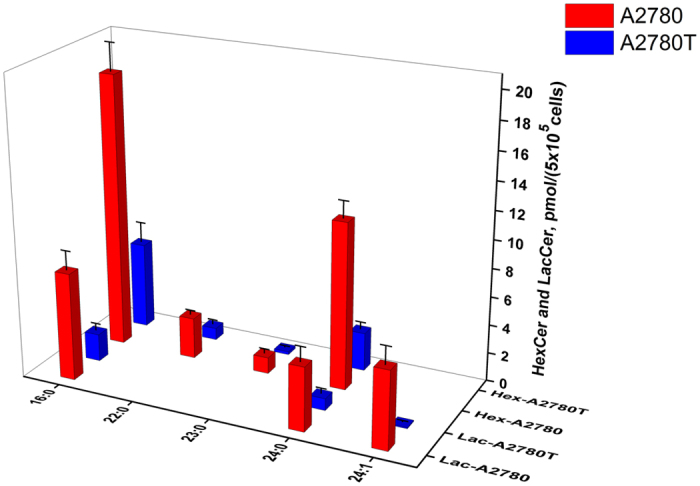
Content of marker HexCer and marker LacCer in A2780 and A2780T. The horizontal axis represents the composition of fatty acid acyl chain of the d18:1 HexCer and d18:1 LacCer.

Among all the HexCers and LacCers, only d18:1 sphingoid base backbone type was found. All the 12 HexCers and LacCers showed higher intensity in A2780 cells than that in A2780T. Sphinganine, as the precursor of DHCer, showed decrease in A2780T. The overall content of sphingosines was similar in both cell types, but the expression of individual sphingosine was quite different. Higher level of Cer1P (d18:0/20:0) was detected in A2780T (data not shown). Figure 5 showed the trends of all 7 marker HexCers and LacCers between A2780 and A2780T.

### Multivariate analyses of the sphingolipidomic data

**Figure 6:**
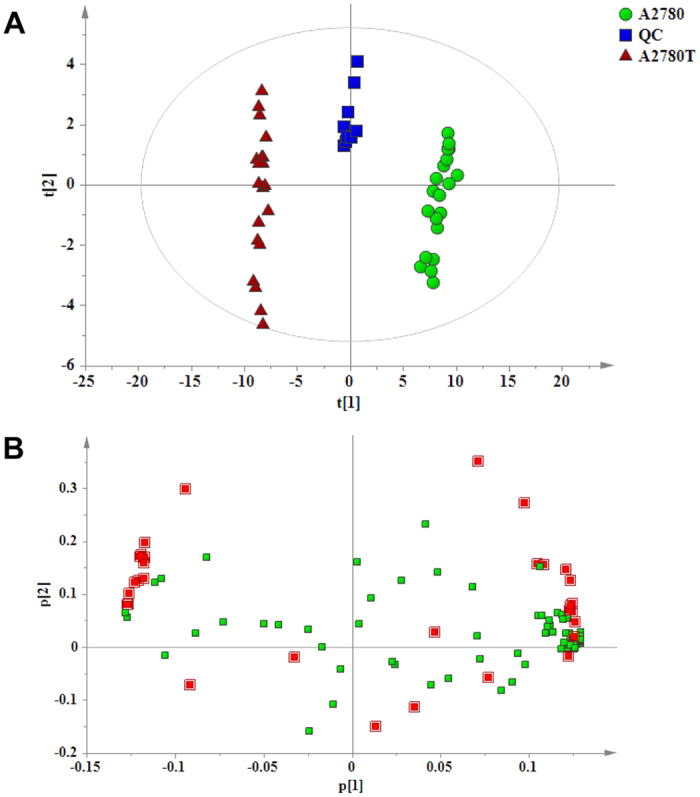
Partial least squares discriminant analysis projecting scatter plots (**A**) scores plot (A2780 & A2780T group, *n* = 20; QC, *n* = 10), (**B**) loading plot. Red boxes represent the SPLs which contribute significantly to the classification (VIP > 1).

Multivariate analyses were further carried out to view the overall differences between A2780 and A2780Ts, and to identify SPL markers that were significantly changed in A2780T. PLS-DA was used to visualize general clustering among A2780, A2780T, and QC groups firstly (Fig. 6A). After auto scaling of data sets, discrimination feature between the profiles were identified for each model by displaying loadings plots. Loading plots and VIP value in PLS-DA model are commonly used for biomarker selection and identification. According to the results, potential SPL markers that were differentially expressed between A2780 and A2780T groups were identified (Fig. 6B and Table 2), suggesting a SPL alternation was involved in A2780T. A total of 52 potential biomarkers were identified according to the VIP value and scattering-plot, among which most of them are sphingomyelins, several highly unsaturated SPLs [SM (d18:1/24:3), SM (d18:1/24:2) and SM (d18:1/22:2)] were also included. LacCer (d18:1/24:1) showed the largest decline in A2780T, whose content decreased by approximately 70 folds, which contributes most significantly to the classification.

## Discussion

In order to drive study on the metabolism of sphingolipids, a reliable and informative analytical method for the comprehensive profiling of SPLs is essential. By using a combined analytical strategy, which enables the reliable identification and sensitive quantification, the dynamic distribution and interconversion of SPLs have been comprehensively monitored. Our improved sphingolipidomic analyses on A2780 and A2780T encompassed most of the important SPLs including sphinganine, sphingosine, ceramide-1-phosphase, hexosylceramide, lactosylceramide, dehydroceramide and dihydroceramide as well dehydrosphingomyelin and dihydrosphingomyelin subclasses. It is the most comprehensive sphingolipidomic study on A2780/A2780T cells to date, as evidenced by the identification of up to 102 SPLs including 67 species that are reported in the cell line for the first time. Distinguished from previous studies, this research of SPL took advantage of a well-established LC-MS method, and looked into the content variation of individual SPL species instead of the overall content of each subclass, thus provided much more detailed and useful information for revealing the mechanism of taxol-resistance. Most of the identified SPLs can be the metabolic pathway related biomarkers, especially the low abundance species of which the subtle changes may result in altered biological function like drug resistance^23^. It’s noted that all the rare SPLs (odd number of carbons/high level of unsaturation) in A2780/A2780T are with the low abundance. Similarly, SPLs with odd number of carbons (C15 and C17) have been reported^24^, and highly unsaturated SPLs were isolated from halotolerant fungus with poor natural abundance^25^. Even in A2780 cell line, Cer with C23 and C25 N-acetyl chain have already been found^19^. Discovery of these rare SPL species is one of the research highlights of this study.

Comparing to taxol sensitive A2780 cells, the most notable alteration in A2780T cells was the overall decrease of Cers. Eleven Cers are recognized as biomarkers of A2780T, along with a 1.5 to 13-fold decrease has been observed. Cer is known as an intracellular messenger that is able to regulate many intracellular effectors mediating activation of the apoptotic process. It has been recognized as a kind of tumor suppressor and has been found to act as a major player in the action of many chemotherapeutic drugs^26^. Dysregulated metabolism of Cers has been identified as a feature of many drug-resistant cancers^27^, as well as in taxol resistant human ovarian cancer cell line CABA-PTX^28^. In A2780T cells the depletion of Cers could potentially help the cells to evade Cer-induced apoptosis, and thereby can be a crucial mechanism responsible for the drug resistance of A2780T.

Totally 34 SMs (including 26 dehydrosphingomyelins and 8 DHSMs) were identified as biomarkers, which took most proportion of the biomarkers. Among all the marker SMs, the content of 6 dehydrosphingomyelins increased in taxol-resistant cells compared to sensitive cells by 0.4–1.2 times. Especially, C16-SMs, a kind of high abundance SPL in both A2780 and A2780T, were found to be significantly higher in the taxol resistant cells than those in the sensitive cells. This leads to the increased total SM level in A2780T, same as previously reported in 2780AD cells^29^. However, the other 20 dehydrosphingomyelin biomarkers decreased by up to 90%. Individually, the content of all d18:2 SMs, d18:1 SMs with unsaturated double bond(s) at the N-acyl chain, as well as d18:1 SMs with saturated N-Acyl chain of C18 to 23 decreased significantly in A2780T comparing to that of A2780. Whilst the content of d18:1 SMs with saturated N-Acyl chain of C16–C17 and C24–C26, as well as all DHSMs, were found to increase significantly in A2780T comparing to A2780. Of note, the increase of DHSMs in A2780T is high up to 8-fold for most species. Dihydrosphingolipids have received increasing attention. Wang *et al*. have determined that 4-HPR treated MDR cancer cells displayed elevations in DHCer but not dehydroceramides, together with elevated DHSM species rather than dehydrosphingomyelins^30^. It indicates that dihydrosphingolipids may fulfil a distinctive role in the metabolic pathway comparing to unsaturated sphingolipids. In A2780T, significant increase of dehydrosphingomyelins and DHSMs concomitant with decrease of corresponding DHCers (which was not identified as markers) have been observed. These variations are consistent with the hypothetical “DHCer - DHSM - dehydrosphingomyelin” pathway, and the activity of related enzymes (dihydroceramide desaturase and dihydroceramide synthase) may be altered^31^.

Cer plays a central role in the sphingolipid metabolism. All the Cers showed consistent trend of decrease in A2780T, except for some extremely low species (DHCers) whose content cannot meet the limit of quantitation. The overall decrease of Cers and accompanying increase of most SMs in A2780T cells, especially, the decrease of two most abundant Cers [Cer (d18:1/24:0) and Cer (d18:1/16:0)] and concomitant increase of corresponding species of SMs [SM (d18:1/24:0) and SM (d18:1/16:0)], clearly indicated SM-related depletion of Cers in A2780T cells. The roles of SMase and SMS in cancer treatment have been well recognized for decades. Their actions have been defined as one of the main routes for the alteration of Cer^8^. Sphingomyelinases are key enzymes of sphingolipid metabolism that regulate the formation and degradation of ceramide^32^. Drugs (including taxol) enhanced ceramide-governed cytotoxic response by activating sphingomyelinase^27^. While SM is the end product in the SM-Cer related pathway, and inhibition of SMS will result in Cer accumulation with effect solely on SM^33^. Thus, it can be proposed that in A2780T cells, the decreased level of Cers might be resulted from the down-regulated expression/activity of SMase or up-regulated SMS expression/activity. Similar mechanism has been reported that a decrease of the ceramide level via activation of glucosylceramide synthase (GCS) and SMS was detected in chemoresistant HL-60/ADR human promyelocytic leukemia cells^34^.

Besides Cer and SM, other SPLs and SPL metabolites also have biological activities that could be responsible for the acquisition of a drug resistance phenotype^35^. Ceramide glycosylation by the enzyme glucosylceramide synthase, which forms glucosylceramide and has been noted in some drug-resistant cell lines, is an important pathway for bypassing apoptosis^36^,^37^. Because SPLs comprised of d18:1 sphingosine backbone are the major species found in mammals^38^, in A2780T only HexCer and LacCer with d18:1 backbone can be detected and further quantified as markers. Additionally, glucosylceramide is known as an intermediate metabolite in the synthesis and degradation of the more complex gangliosides, and a number of drug-resistant cancer cell lines accumulate this noncytotoxic metabolite^27^. In our case of A2780T, the decrease of glucosylceramide and LacCer can be explained as “activation of ganglioside pathway”^8^, which enable cancer cells convert Cer into gangliosides to evade the pro-apoptotic function of Cer. The enzymes related to the “glucosylceramide-lactosylceramide-ganglioside pathway”, including glucosylceramidase, glucosylceramide synthase, and lactosylceramide synthase, could have participated in the biological progress.

In A2780T, reduced syntheses of Cer, HexCer, and LacCer were observed, with the concomitant increase of DHSM and total SM levels, in which C16-SMs contributes the vast majority. These represent the main sphingolipid metabolism pattern in A2780T, which is significantly different from the SPL profiles in similar ovarian cancer cell lines. On one hand, comparing with the sphingolipidome in another taxol resistant human ovarian cell line CABA-PTX^28^, the level of sphingomyelin in A2780T changed significantly. On the other hand, A2780T also showed different sphingolipidomic profile from A2780 cell lines resistant to other drugs. In sharp contrast to the well-studied MDR A2780 cells^29^, the rise of cellular HexCer (including galactosylceramide and glucosylcermide) levels was not observed in A2780T. And in A2780/HPR cells the glycosphigolipid-dominated alteration^20^ is also different from the SPL pattern in A2780T, which possesses a distinctive feature of “SM-related depletion of Cers”. It indicated that the resistant mechanism of A2780T could be different from that of either other taxol-resistant cancer cells (CABA-PTX) or A2780 cells resistant to other drugs (MDR A2780 & A2780/HPR). Such interdisciplinary basic scientific research has close relevance to the medical community and it facilitates the applications in rapid detection and classification of disease type (taxol-resistant or not) and medication direction.

## Conclusions

Since the role of sphingolipids in cancer cell has been widely recognized, comprehensive sphingolipidomic study is essential for exploring its drug resistance mechanism. The most comprehensive and accurate method described in this paper fully identified SPLs in A2780 human ovarian cancer cell line and the taxol-resistant cell line A2780T. Most individual species, including some low abundance but biologically important SPLs, have been accurately quantified. It provides more detailed information than general overview of a whole subclass, which is significant for studying the alterations^39^.

The sphingolipid metabolism in A2780T is oriented toward down-regulation of ceramides. We propose A2780T cells may escape from the ceramide-caused apoptosis mainly via sphingomyelin/ceramide pathway, while SMS was expressed more in A2780T than in the sensitive cell line, or the activity of SMase was inhibited. These enzymes related to the marker SPLs and altered pathways, are the potential targets. Based on the sphingolipidomic study, adjusting the sphingolipid metabolism purposively may represent a winning strategy to overcome taxol resistance and improve cancer therapy. This study facilitates not only development of new drugs against taxol resistance, but also clinical diagnosis of taxol-resistant ovarian cancer.

## Methods

### Chemicals and solutions

Human ovarian cancer cell line (A2780) and its taxol-resistant strain (A2780T) were purchased from KeyGen Biotech Co., Ltd. (Nanjing, China). The LIPID MAPS internal standard (IS) cocktail in ethanol, composed of 25 μM each of nine sphingolipid standards including SM (d18:1/12:0), Cer (d18:1/12:0), C1P (d18:1/12:0), HexCer (d18:1/12:0), LacCer (d18:1/12:0), Sphinganine (d17:0), Sphingosine (d17:1), Sphinganine-1-Phosphate (d17:0) and Sphingosine-1-Phosphae (d17:1), was purchased from Avanti Polar Lipids (Alabaster, AL, USA). HPLC-grade methanol (MeOH), chloroform (CHCl_3_) and isopropanol (IPA) were purchased from Merck (Darmstadt, Germany). Ammonium acetate (NH_4_OAc), potassium hydroxide (KOH), acetic acid (CH_3_COOH) and formic acid (HCOOH) were purchased from Sigma-Aldrich (St. Louis, MO, USA). Dulbecco’s Modified Eagle’s Medium (DMEM), Roswell Park Memorial Institute (RPMI) 1640 medium, Fetal Bovine Serum (FBS), Penicillin-Streptomycin (PS) were purchased from Gibco, New Zealand. Sodium dodecyl sulfate (SDS) and 3-(4,5-dimethylthiazol-2-yl)-2,5-diphenyltetrazolium bromide (MTT) were purchased from Acros, USA.

A pooled sample equally aliquoted from all samples can provide the most comprehensive information within a specific study. Hence, equivalent amount of A2780T was spiked into A2780 to prepare a pooled quality control (QC) sample.

### Cell culture and SPLs Extraction

A2780 human ovarian cancer cell line were cultured in DMEM supplemented with 1% PS and 10% FBS in a humidified 5% CO_2_ atmosphere at 37 °C. For lipid analysis, cells were seeded into dishes and grown to confluence. Cells were rinsed twice with ice-cold PBS and scraped into a borosilicate glass tube with polytetrafluoroethylene coated top. After adding 0.5 mL of MeOH, 0.25 mL of CHCl_3_ and 10 μL of internal standards cocktail (2.5 μM) successively, the mixture was sonicated at room temperature for 30 s then incubated at 48 °C for 12 h to extract SPLs. After 75 μL of KOH in MeOH (1M) was added in, the mixture was incubated in a shaking water bath for 2 h at 37 °C to cleave potentially interfering glycerolipids. After cooling and neutralization with acetic acid, a four-step extraction procedure was performed as reported to prepare the SPLs for LC-MS analysis.

In order to verify the taxol resistance in the commercial A2780T cell line, MTT assay was employed. A2780T cells were cultured in RPMI 1640 medium containing 10% FBS, 1% PS and 800 ng/mL taxol solution at 37 °C in a 5% CO_2_/95% air atmosphere. For assessment of cell viability, A2780 and A2780T were respectively seeded in a 96-well plate at a density of 5 × 10^3^ cells/well and were allowed to adhere for 16 h before treatment. Following incubation for 48 h, MTT solution (10 μL per well, 5 mg/mL solution) was added to each well and incubated for 4 h at 37 °C. Thereafter 100 μL 10% SDS was added for lysing and the plate was maintained overnight at 37 °C in a 5% CO_2_/95% air atmosphere. The optical densities were determined at 570 nm using a microplate reader. The procedure was repeated three times.

The sensitivity of A2780 and A2780T cell lines to taxol was assayed by using MTT assay. The IC_50_ for taxol in A2780 and A2780T was 73.16 nM and 149.6 μM, respectively. The result indicates that resistance to taxol of A2780T is at least 1000 fold greater than that of A2780.

### LC-MS conditions

Sphingolipid analysis was performed by using our well-established LC-MS method^21^ with minor optimization, an Agilent 1290 UHPLC tandem 6550 quadrupole time-of-flight (Q-TOF) MS system and an Agilent 1290 UHPLC tandem 6460 triple quadrupole (QQQ) MS system were employed for qualitative profiling and quantitative analysis respectively. Chromatographic separation was performed as described previously, while the injection volume was 10 μL for Q-TOF and 5 μL for QQQ, respectively. An Agilent Eclipse Plus C_18_ column (100 × 2.1 mm, 1.8 μm) was used to separate the endogenous SPLs. The mobile phase consisted of (A) MeOH/H_2_O/HCOOH (60:40:0.2, *v*/*v*/*v*) and (B) MeOH/IPA/HCOOH (60:40:0.2, *v*/*v*/*v*), both containing 10 mM NH_4_OAc. The flow rate was 0.4 mL/min, and the column temperature was maintained at 40 °C for each run. A linear gradient was optimized as follows: 0–3 min, 0% to 10% B; 3–5 min, 10% to 40% B; 5–5.3 min, 40% to 55% B; 5.3–8 min, 55% to 60% B; 8–8.5 min, 60% to 80% B; 8.5–10.5 min, 80% to 80% B; 10.5–16 min, 80% to 90% B; 16–19 min, 90% to 90% B; 19–22 min, 90% to 100% B, followed by washing with 100% B and equilibration with 0% B. A typical data acquisition time was 20 min.

The above UHPLC system was interfaced with an Agilent ultrahigh definition (UHD) 6550 Q-TOF mass spectrometer equipped with an ESI source (Santa Clara, CA, USA). The source parameters were: drying gas (N_2_) temperature 150 °C, flow rate 15 L/min, nebulizer pressure 25 psi, sheath gas (N_2_) temperature was set at 200 °C with a flow-rate at 12 L/min. The scan parameters were: positive ion mode over *m*/*z* 110–1200, capillary voltage 4000 V, nozzle voltage 300 V, fragmentor voltage 175 V, skimmer voltage 65 V, octopole RF peak 500 V, drying gas 6 L/min at 300 °C. A reference solution was nebulized for continuous calibration in positive ion mode using the reference mass *m*/*z* 922.00979800. The acquisition and data analysis were controlled using Agilent Mass Hunter Workstation Software (Agilent, USA).

The UHPLC conditions for quantitative analysis were the same as those mentioned above. The LC system was coupled to an Agilent 6460 triple-quadrupole mass spectrometer (Santa Clara, CA, USA). The ESI parameters were optimized as follows: positive ion mode, drying gas (N_2_) temperature 325 °C, flow rate 11 L/min, nebulizer pressure 45 psi, capillary voltage 4000 V, nozzle voltage 300 V, sheath gas (N_2_) temperature was set at 200 °C with a flow-rate at 12 L/min. Data were processed with Agilent Mass Hunter Workstation Software. Further detail of the parameters, such as characteristic transitions, fragmentor and CE voltages optimized for each compound, and the methodology validations are similar as described before.

### Data analysis

The screening and identification of SPLs were performed by searching in our personal database, which was built and updated based on the Agilent Mass Hunter Personal Compound Database and Library (PCDL) software and LIPID MAPS information (31643 SPLs until July 08 2015).

The sphingolipidomic approach was applied in qualitative research of SPLs by analyzing a pooled sample equally mixed by A2780 and A2780T. In quantitative research, A2780 cells (models, *n* = 10) and A2780T cells (models, *n* = 10) were analyzed in parallel. Supervised multivariate statistical analysis, partial least squares to latent structure-discriminant analysis (PLS-DA) method, was used to differentiate the amounts of SPLs between the two strains. Potential biomarkers were selected according to Variable Importance in Projection (VIP) value and the loading scattering-plot, using SIMCA-P+ software version 13.0 (Umetrics, Umea, Sweden). VIP values higher than 1.00 were considered significant.

## Additional Information

**How to cite this article**: Huang, H. *et al*. LC-MS Based Sphingolipidomic Study on A2780 Human Ovarian Cancer Cell Line and Its Taxol-resistant Strain. *Sci. Rep.* **6**, 34684; doi: 10.1038/srep34684 (2016).

## Figures and Tables

**Table 1:** Identification and quantification of SPLs in A2780/A2780T cells by using UHPLC-Q-TOF and UHPLC-QQQ MS.

Class	Name	[M + H]^+^ *m*/*z*	t_R_ (min)	Molecular Formula	Measured Mass	Calculated Mass	Error (ppm)	MS/MS Fragments (*m*/*z*)	MRM transitions	
SM	d18:1/26:0	843.7315	18.391	C_49_ H_99_ N_2_ O_6_ P	842.7243	842.7241	0.23	264.2693, 184.0732	843.8	184.1
	d18:1/26:1	841.7088	17.057	C_49_ H_97_ N_2_ O_6_ P	840.7046	840.7084	−4.52	264.2692, 184.0735	841.7	184.1
	d18:1/25:0	829.7152	17.641	C_48_ H_97_ N_2_ O_6_ P	828.7085	828.7084	0.14	264.2638, 184.0733	829.7	184.1
	d18:1/25:1	827.6987	16.441	C_48_ H_95_ N_2_ O_6_ P	826.6898	826.6928	−3.59	264.2655, 184.0736	827.7	184.1
	d18:1/24:0	815.7009	16.974	C_47_ H_95_ N_2_ O_6_ P	814.6936	814.6928	1.01	264.2668, 184.0730	815.7	184.1
	d18:1/24:1 **[A**_**1**_]	813.6851	15.807	C_47_ H_93_ N_2_ O_6_ P	812.6780	812.6771	1.01	264.2697, 184.0735	813.7	184.1
	d18:1/24:2	811.6692	14.907	C_47_ H_91_ N_2_ O_6_ P	810.6618	810.6615	0.35	264.2702, 184.0734	811.7	184.1
	d18:1/24:3	809.6528	14.173	C_47_ H_89_ N_2_ O_6_ P	808.6456	808.6458	−0.3	264.2669, 184.0732	809.6	184.1
	d18:1/23:0	801.6848	16.324	C_46_ H_93_ N_2_ O_6_ P	800.6774	800.6771	0.37	264.2674, 184.0731	801.7	184.1
	d18:1/23:1 **[A**_**2**_]	799.6689	15.174	C_46_ H_91_ N_2_ O_6_ P	798.6615	798.6615	0.01	282.2457, 264.2695, 184.0731	799.7	184.1
	d18:1/23:2	797.6507	14.273	C_46_ H_89_ N_2_ O_6_ P	796.6436	796.6458	−2.84	264.2667, 184.0731	797.6	184.1
	d18:1/22:0	787.6692	15.674	C_45_ H_91_ N_2_ O_6_ P	786.6617	786.6615	0.32	264.2655, 184.0733	787.7	184.1
	d18:1/22:1 **[A**_**3**_]	785.6533	14.574	C_45_ H_89_ N_2_ O_6_ P	784.6455	784.6458	−0.45	264.2688, 184.0731	785.7	184.1
	d18:1/22:2	783.6374	13.690	C_45_ H_87_ N_2_ O_6_ P	782.6291	782.6302	−1.32	264.2700, 184.0726	783.7	184.1
	d18:1/21:0	773.6527	15.057	C_44_ H_89_ N_2_ O_6_ P	772.6453	772.6458	−0.68	264.2674, 184.0729	773.7	184.1
	d18:1/21:1	771.6340	14.123	C_44_ H_87_ N_2_ O_6_ P	770.6270	770.6302	−4.13	264.2679, 184.0728	771.7	184.1
	d18:1/20:0	759.6372	14.390	C_43_ H_87_ N_2_ O_6_ P	758.6300	758.6302	−0.21	264.2734, 184.0731	759.7	184.1
	d18:1/19:0	745.6213	13.757	C_42_ H_85_ N_2_ O_6_ P	744.6138	744.6145	−0.95	264.2689, 184.0726	745.7	184.1
	d18:1/18:0	731.6068	13.140	C_41_ H_83_ N_2_ O_6_ P	730.6005	730.5989	2.16	264.2678, 184.0731	731.6	184.1
	d18:1/18:1	729.5906	12.323	C_41_ H_81_ N_2_ O_6_ P	728.5827	728.5832	−0.75	264.2699, 184.0732	729.6	184.1
	d18:1/17:0	717.5911	12.573	C_40_ H_81_ N_2_ O_6_ P	716.5841	716.5832	1.17	264.2622, 184.0731	717.6	184.1
	d18:1/16:0	703.5754	12.023	C_39_ H_79_ N_2_ O_6_ P	702.5684	702.5676	1.11	264.2694, 184.0731	703.6	184.1
	d18:1/16:1	701.5604	11.323	C_39_ H_77_ N_2_ O_6_ P	700.5530	700.5519	1.53	264.2645, 184.0732	701.6	184.1
	d18:1/15:0	689.5595	11.573	C_38_ H_77_ N_2_ O_6_ P	688.5521	688.5519	0.27	264.2750, 184.0732	689.6	184.1
	d18:1/14:0	675.5427	11.190	C_37_ H_75_ N_2_ O_6_ P	674.5369	674.5363	0.96	264.2676, 184.0732	675.5	184.1
	d18:2/24:0 **[A**_**1**_]	813.6848	16.074	C_47_ H_93_ N_2_ O_6_ P	812.6774	812.6771	0.33	262.2513, 184.0730	813.7	184.1
	d18:2/24:3	807.6350	14.590	C_47_ H_87_ N_2_ O_6_ P	806.6291	806.6302	−1.29	262.2523, 184.0731	807.6	184.1
	d18:2/23:0 **[A**_**2**_]	799.6690	15.424	C_46_ H_91_ N_2_ O_6_ P	798.6615	798.6615	0.09	262.2512, 184.0732	799.7	184.1
	d18:2/22:0 **[A**_**3**_]	785.6531	14.757	C_45_ H_89_ N_2_ O_6_ P	784.6458	784.6458	−0.07	262.2620, 184.0730	785.7	184.1
	d18:2/20:0	757.6213	13.490	C_43_ H_85_ N_2_ O_6_ P	756.6138	756.6145	−0.93	262.2523, 184.0730	757.7	184.1
	d18:2/15:0	687.5433	10.906	C_38_ H_75_ N_2_ O_6_ P	686.5355	686.5363	−1.17	262.2503, 184.0716	687.5	184.1
	d18:1/12:0 **[IS-1]**	647.5133	10.389	C_35_ H_71_ N_2_ O_6_ P	646.5058	646.505	1.32	264.2699, 184.0732	647.5	184.1
DHSM	d18:0/25:0	831.7299	18.324	C_48_ H_99_ N_2_ O_6_ P	830.7233	830.7241	−0.89	184.0745		
	d18:0/24:0	817.7156	17.524	C_47_ H_97_ N_2_ O_6_ P	816.7082	816.7084	−0.3	184.0726	817.7	184.1
	d18:0/23:0	803.6991	16.874	C_46_ H_95_ N_2_ O_6_ P	802.6919	802.6928	−1.14	266.2781, 184.0724	803.7	184.1
	d18:0/22:0	789.6843	16.207	C_45_ H_93_ N_2_ O_6_ P	788.6769	788.6771	−0.31	184.0734	789.7	184.1
	d18:0/20:0	761.6530	14.907	C_43_ H_89_ N_2_ O_6_ P	760.6456	760.6458	−0.25	184.0724	761.7	184.1
	d18:0/19:0	747.6388	14.373	C_42_ H_87_ N_2_ O_6_ P	746.6310	746.6302	1.08	184.0727	747.6	184.1
	d18:0/18:0	733.6222	13.64	C_41_ H_85_ N_2_ O_6_ P	732.6176	732.6145	4.15	184.0731	733.7	184.1
	d18:0/17:0	719.5692	10.673	C_39_ H_79_ N_2_ O_7_ P	718.5617	718.5625	−1.06	184.0725	719.6	184.1
	d18:0/16:0	705.5915	12.457	C_39_ H_81_ N_2_ O_6_ P	704.5843	704.5832	1.58	184.0735	705.6	184.1
	d18:0/15:0	691.5747	11.940	C_38_ H_79_ N_2_ O_6_ P	690.5685	690.5676	1.35	184.0731	691.6	184.1
	d18:0/14:0	677.5576	11.523	C_37_ H_77_ N_2_ O_6_ P	676.5519	676.5519	0.01	184.0729	677.5	184.1
	t18:0/16:0	721.5839	11.423	C_39_ H_81_ N_2_ O_7_ P	720.5769	720.5781	−1.74	264.2685, 184.0719	721.6	184.1
Cer	d18:1/24:0	650.6451	18.458	C_42_ H_83_ N O_3_	649.6379	649.6373	0.86	632.6290, 614.6156, 264.2683	650.7	264.3
	d18:1/24:1	648.6293	17.157	C_42_ H_81_ N O_3_	647.6220	647.6216	0.62	630.6170, 612.6100, 264.2690	648.7	264.3
	d18:1/24:2	646.6124	16.224	C_42_ H_79_ N O_3_	645.6058	645.6060	−0.25	264.2703	646.7	264.3
	d18:1/23:0(OH)	652.6250	16.194	C_41_ H_81_ N O_4_	651.6185	651.6166	0.94	264.2697	652.7	264.3
	d18:1/23:0	636.6288	17.707	C_41_ H_81_ N O_3_	635.6213	635.6216	−0.48	264.2689	636.6	264.3
	d18:1/23:1	634.6135	16.507	C_41_ H_79_ N O_3_	633.6055	633.6060	−0.73	264.2670	634.6	264.3
	d18:1/22:0	622.6136	17.007	C_40_ H_79_ N O_3_	621.6077	621.6060	2.77	264.2700	622.6	264.3
	d18:1/22:1 **[A**_**4**_]	620.5983	15.874	C_40_ H_77_ N O_3_	619.5893	619.5903	−1.74	264.2684	620.5	264.3
	d18:1/20:0	594.5808	15.600	C_38_ H_75_ N O_3_	593.5735	593.5747	−1.22	264.2674	594.6	264.3
	d18:1/18:0	566.5521	14.340	C_36_ H_71_ N O_3_	565.5440	565.5434	1.15	264.2681	566.6	264.3
	d18:1/18:1	564.5332	13.167	C_36_ H_69_ N O_3_	563.5251	563.5277	−4.62	264.2660	564.5	264.3
	d18:1/17:3	546.4890	10.890	C_35_ H_63_ N O_3_	545.4819	545.4808	2.1	264.2701		
	d18:1/16:0	538.5198	13.057	C_34_ H_67_ N O_3_	537.5124	537.5121	0.57	264.2684	538.5	264.3
	d18:1/16:1	536.5045	12.243	C_34_ H_65_ N O_3_	535.4974	535.4964	1.74	264.2694	536.6	264.3
	d18:1/15:3(OH)	534.4521	13.906	C_33_ H_59_ N O_4_	533.4449	533.4444	0.98	516.4403, 264.2706	534.5	264.3
	d18:1/14:3(OH)	520.4367	13.473	C_32_ H_57_ N O_4_	519.4297	519.4288	1.81	502.4256, 264.2679	520.4	264.3
	d18:2/23:1	632.5940	15.657	C_41_ H_77_ N O_3_	631.5885	631.5903	−2.86	262.2520	632.6	262.3
	d18:2/22:0 **[A**_**4**_]	620.5979	16.074	C_40_ H_77_ N O_3_	619.5906	619.5903	0.45	602.5864, 262.2610	620.5	262.3
	d17:1/16:0	524.5045	12.477	C_33_ H_65_ N O_3_	523.4971	523.4964	1.27	250.2520	524.5	250.3
	d18:1/12:0 **[IS-2]**	482.4574	10.990	C_30_ H_59_ N O_3_	481.4501	481.4495	1.3	264.2678	482.5	264.3
DHCer	d18:0/24:0	652.6607	19.112	C_42_ H_85_ N O_3_	651.6533	651.6529	0.57	634.6377, 266.2767	652.7	266.3
	d18:0/18:3	562.5197	15.924	C_36_ H_67_ N O_3_	561.5122	561.5121	0.25	266.2797		
	d18:0/16:0	540.5354	13.507	C_34_ H_69_ N O_3_	539.5273	539.5277	−0.86	266.2833	540.5	266.3
	d18:0/17:0(OH)	570.5458	13.408	C_35_ H_71_ N O_4_	569.5383	569.5383	0.05	266.2642		
	d18:0/16:0(OH)	556.5300	12.373	C_34_ H_69_ N O_4_	555.5227	555.5227	0.05	266.2857		
	d18:0/14:0(OH)	528.4993	11.256	C_32_ H_65_ N O_4_	527.4921	527.4914	1.49	266.2831		
	d17:0/13:0(OH)	500.4682	11.323	C_30_ H_61_ N O_4_	499.4608	499.4601	1.5	252.2673		
C1P	d18:1/19:0(OH)	676.5279	11.823	C_37_ H_74_ N O_7_ P	675.5200	675.5203	−0.37	264.2673	676.5	264.3
	d18:1/12:2	558.3904	9.573	C_30_ H_56_ N O_6_ P	557.3832	557.3845	−2.44	264.2677		
	d18:1/12:0 **[IS-3]**	562.4223	10.006	C_30_ H_60_ N O_6_ P	561.4149	561.4158	−1.72	264.2688	562.5	264.3
HexCer	d18:1/26:0	840.7282	18.374	C_50_ H_97_ N O_8_	839.7213	839.7214	−0.11	264.2774	840.7	264.3
	d18:1/24:0	812.6975	16.957	C_48_ H_93_ N O_8_	811.6901	811.6901	−0.07	632.6302, 264.2684	812.7	264.3
	d18:1/24:1	810.6800	15.807	C_48_ H_91_ N O_8_	809.6728	809.6745	−2.01	630.6137, 264.2676	810.6	264.3
	d18:1/23:0	798.6816	16.307	C_47_ H_91_ N O_8_	797.6738	797.6745	−0.85	618.6106, 264.2682	798.7	264.3
	d18:1/22:0	784.6656	15.674	C_46_ H_89_ N O_8_	783.6580	783.6588	−1.02	604.6012, 264.2689	784.7	264.3
	d18:1/20:1	754.6178	13.173	C_44_ H_83_ N O_8_	753.6105	753.6119	−1.77	264.2691	754.6	264.3
	d18:1/16:0	700.5722	12.040	C_40_ H_77_ N O_8_	699.5645	699.5649	−0.6	264.2694	700.6	264.3
	d18:1/12:0 **[IS-4]**	644.5101	10.406	C_36_ H_69_ N O_8_	643.5028	643.5023	0.78	264.2684	644.5	264.3
LacCer	d18:1/24:0	974.7501	16.324	C_54_ H_103_ N O_13_	973.7440	973.7429	1.05	794.6828, 614.6288, 264.2674	974.8	264.3
	d18:1/24:1	972.7323	15.19	C_54_ H_101_ N O_13_	971.7247	971.7273	−2.65	264.2694	972.8	264.3
	d18:1/22:0	946.7175	15.057	C_52_ H_99_ N O_13_	945.7100	945.7116	−1.7	766.3514, 264.2723	946.8	264.3
	d18:1/20:0	918.6891	13.990	C_50_ H_95_ N O_13_	917.6815	917.6803	1.22	264.2665		
	d18:1/18:0	890.6541	12.660	C_48_ H_91_ N O_13_	889.6463	889.6490	−3.07		890.7	264.3
	d18:1/16:0	862.6245	11.623	C_46_ H_87_ N O_13_	861.6175	861.6177	−0.26	844.6130, 520.5112, 502.4944, 264.2688	862.7	264.3
	d18:1/12:0 **[IS-5]**	806.5624	10.189	C_42_ H_79_ N O_13_	805.5549	805.5551	−0.26	464.4472, 264.2683	806.7	264.3
Sa	d19:0	316.3202	6.532	C_19_ H_41_ N O_2_	315.3141	315.3137	1.23	298.3106, 280.2984	316.3	298.3
	d16:0	274.2742	4.842	C_16_ H_35_ N O_2_	273.2669	273.2668	0.43	256.2604	274.3	256.3
	t19:0	332.3166	6.691	C_19_ H_41_ N O_3_	331.3093	331.3086	1.87	314.3032, 296.2776	332.3	314.3
	t17:0	304.2855	5.841	C_17_ H_37_ N O_3_	303.2782	303.2773	2.68	286.2729		
	Capnine	352.2519	5.075	C_17_ H_37_ N O_4_ S	351.2445	351.2443	0.47	316.1840		
	Enigmol	302.3050	6.765	C_18_ H_39_ N O_2_	301.2978	301.2981	−1.66	284.2934	302.4	284.3
	Xestoaminol C	230.2481	2.060	C_14_ H_31_ N O	229.2408	229.2406	1.01	212.2380	230.3	212.3
	d17:0 **[IS-6]**	288.2901	6.632	C_17_ H_37_ N O_2_	287.2829	287.2824	1.65	270.2794	288.3	270.3
So	d19:1	314.3056	10.673	C_19_ H_39_ N O_2_	313.2983	313.2981	0.69	296.3320	314.3	296.3
	d18:1	300.2898	6.741	C_18_ H_37_ N O_2_	299.2824	299.2824	−0.08	282.2780, 264.2669	300.3	282.3
	d16:1	272.2582	5.418	C_16_ H_33_ N O_2_	271.2505	271.2511	−2.36	254.2849	272.3	254.2
	d16:3	268.2277	6.458	C_16_ H_29_ N O_2_	267.2203	267.2198	1.82	250.2529, 238.2509		
	d15:3	254.2117	4.358	C_15_ H_27_ N O_2_	253.2045	253.2042	1.08	236.1994		
	t19:1	330.3009	6.405	C_19_ H_39_ N O_3_	329.2936	329.293	1.9	312.3288	330.3	312.3
	t19:2	328.2852	8.274	C_19_ H_37_ N O_3_	327.2777	327.2773	1.13	310.3129	328.3	310.3
	t18:1	316.2850	6.874	C_18_ H_37_ N O_3_	315.2776	315.2773	0.88	298.2911	316.3	298.3
	t17:1	302.2696	7.024	C_17_ H_35_ N O_3_	301.2624	301.2617	2.21	284.2947	302.3	284.3
	N,N-dimethylSo	328.3210	9.473	C_20_ H_41_ N O_2_	327.3136	327.3137	−0.24	310.3091	328.3	310.3
	Halaminol A	228.2319	6.891	C_14_ H_29_ N O	227.2246	227.2249	−1.17	210.0276	228.2	210.0
	d17:1 **[IS-7]**	286.3106	6.558	C_18_ H_39_ N O	285.3034	285.3032	0.74	268.2643	286.3	268.3
Sa1P	d17:0 **[IS-8]**	368.2574	6.774	C_17_ H_38_ N O_5_ P	367.2504	367.2488	4.57		368.3	270.3
So1P	d17:1 **[IS-9]**	366.2406	6.558	C_17_ H_36_ N O_5_ P	365.2331	365.2331	0.03	250.2510	366.2	250.3

SM, sphingomyelin; DHSM, dihydrosphingomyelin; Cer, Ceramide; DHCer, dihydroceramide; HexCer, hexosylceramide; LacCer, lactosylceramide; Sa, sphinganine; So, sphingosine; C1P, ceramide-1-phosphate; Sa1P, sphinganine-1-phosphate; So1P, sphingosine-1-phosphate. [**A**_**1**_**–A**_**4**_], 4 pairs of isomeric sphingolipids; [**IS]**, internal standard.

**Table 2:** Quantification of SPLs (VIP > 1) in A2780 and A2780T.

SPLs	Content (pmol/5*10^5^ cells)		Change A2780T vs A2780	*p* value	VIP
	A2780 (*n* = 10)	A2780T (*n* = 10)			
SM(d18:2/23:0)	1.91 ± 0.06	1.15 ± 0.03	↓	<0.001	1.09801
SM(d18:2/22:0)	5.68 ± 0.16	3.19 ± 0.09	↓	<0.001	1.13525
SM(d18:2/20:0)	1.14 ± 0.03	0.30 ± 0.02	↓	<0.001	1.16777
SM(d18:1/26:1)	1.23 ± 0.04	0.57 ± 0.02	↓	<0.001	1.13507
SM(d18:1/26:0)	0.39 ± 0.01	0.85 ± 0.02	↑	<0.001	1.15741
SM(d18:1/25:1)	1.09 ± 0.03	0.81 ± 0.02	↓	<0.001	1.01815
SM(d18:1/25:0)	0.69 ± 01	1.16 ± 0.02	↑	<0.001	1.11518
SM(d18:1/24:3)	0.85 ± 0.04	0.21 ± 0.01	↓	<0.001	1.14943
SM(d18:1/24:2)	22.5 ± 0.42	4.00 ± 0.09	↓	<0.001	1.18383
SM(d18:1/24:1)	94.6 ± 1.80	22.5 ± 0.65	↓	<0.001	1.18238
SM(d18:1/24:0)	35.6 ± 0.64	55.1 ± 1.33	↑	<0.001	1.13437
SM(d18:1/23:2)	0.72 ± 0.02	0.04 ± 0.00	↓	<0.001	1.15961
SM(d18:1/23:1)	8.43 ± 0.18	1.27 ± 0.04	↓	<0.001	1.18228
SM(d18:1/22:2)	0.49 ± 0.03	0.08 ± 0.01	↓	<0.001	1.13820
SM(d18:1/22:1)	8.00 ± 0.16	0.84 ± 0.03	↓	<0.001	1.18362
SM(d18:1/21:1)	0.32 ± 0.01	0.05 ± 0.00	↓	<0.001	1.14304
SM(d18:1/21:0)	1.60 ± 0.04	0.62 ± 0.02	↓	<0.001	1.16306
SM(d18:1/20:0)	3.89 ± 0.09	2.02 ± 0.07	↓	<0.001	1.15126
SM(d18:1/19:0)	0.54 ± 0.03	0.15 ± 0.01	↓	<0.001	1.11029
SM(d18:1/18:1)	31.7 ± 0.03	5.77 ± 0.01	↓	<0.001	1.18432
SM(d18:1/18:0)	28.7 ± 0.48	7.54 ± 0.31	↓	<0.001	1.18301
SM(d18:1/17:0)	4.63 ± 0.12	7.27 ± 0.20	↑	<0.001	1.11640
SM(d18:1/16:1)	27.3 ± 0.47	40.1 ± 0.91	↑	<0.001	1.12871
SM(d18:1/16:0)	431 ± 20.9	606 ± 40.4	↑	<0.001	1.11932
SM(d18:1/15:0)	20.0 ± 0.32	14.1 ± 0.26	↓	<0.001	1.14185
SM(d18:1/14:0)	20.9 ± 0.28	15.6 ± 0.27	↓	<0.001	1.13645
DHSM(d18:0/24:0)	0.54 ± 0.01	4.96 ± 0.13	↑	<0.001	1.18082
DHSM(d18:0/23:0)	0.11 ± 0.00	0.81 ± 0.02	↑	<0.001	1.17627
DHSM(d18:0/22:0)	0.74 ± 0.02	3.35 ± 0.08	↑	<0.001	1.17865
DHSM(d18:0/20:0)	0.27 ± 0.01	0.49 ± 0.02	↑	<0.001	1.04670
DHSM(d18:0/18:0)	1.06 ± 0.03	1.87 ± 0.06	↑	<0.001	1.12766
DHSM(d18:0/16:0)	30.1 ± 0.70	198 ± 5.28	↑	<0.001	1.17911
DHSM(d18:0/14:0)	0.72 ± 0.02	2.59 ± 0.08	↑	<0.001	1.17037
DHSM(t18:0/16:0)	0.11 ± 0.01	0.27 ± 0.03	↑	<0.001	1.07421
Cer(d18:2/22:0)	1.07 ± 0.08	0.23 ± 0.04	↓	<0.001	1.09314
Cer(d18:1/24:2)	0.24 ± 0.03	0.02 ± 0.00	↓	<0.001	1.02636
Cer(d18:1/24:1)	14.6 ± 0.59	1.60 ± 0.07	↓	<0.001	1.17972
Cer(d18:1/24:0)	14.6 ± 0.27	5.97 ± 0.20	↓	<0.001	1.17416
Cer(d18:1/23:1)	0.26 ± 0.03	0.02 ± 0.00	↓	<0.001	1.02345
Cer(d18:1/23:0)	1.02 ± 0.04	0.42 ± 0.03	↓	<0.001	1.12215
Cer(d18:1/23:0(OH))	0.48 ± 0.03	0.22 ± 0.02	↓	<0.001	1.02341
Cer(d18:1/22:0)	2.23 ± 0.10	1.16 ± 0.08	↓	<0.001	1.06470
Cer(d18:1/18:1)	6.39 ± 0.23	4.09 ± 0.25	↓	<0.001	1.01738
Cer(d18:1/18:0)	1.62 ± 0.13	0.20 ± 0.04	↓	<0.001	1.10112
Cer(d17:1/16:0)	2.24 ± 0.12	0.47 ± 0.06	↓	<0.001	1.13502
HexCer(d18:1/24:0)	11.7 ± 0.40	2.70 ± 0.18	↓	<0.001	1.16555
HexCer(d18:1/23:0)	1.14 ± 0.40	0.21 ± 0.18	↓	<0.001	1.04684
HexCer(d18:1/22:0)	2.89 ± 0.11	0.85 ± 0.11	↓	<0.001	1.12835
HexCer(d18:1/16:0)	19.3 ± 0.67	6.11 ± 0.49	↓	<0.001	1.15137
LacCer(d18:1/24:1)	5.48 ± 0.43	0.08 ± 0.01	↓	<0.001	1.12942
LacCer(d18:1/24:0)	4.49 ± 0.36	0.84 ± 0.12	↓	<0.001	1.09150
LacCer(d18:1/16:0)	7.59 ± 0.45	1.93 ± 0.17	↓	<0.001	1.11980
